# Rapid Cold Fixation of Mouse Colon (Col’RFix) Enables High-Resolution Mass Spectrometry Imaging

**DOI:** 10.1021/acs.analchem.5c04968

**Published:** 2026-04-13

**Authors:** Sabina H. Skov, Henrik M. Jensen, Ole N. Jensen

**Affiliations:** † Department of Biochemistry and Molecular Biology, University of Southern Denmark, DK-5230 Odense M, Denmark; ‡ Advanced Analytical R&D, Health & Biosciences, 165097 International N&H Denmark ApS (IFF), DK-8220 Brabrand, Denmark

## Abstract

Cellular-scale MALDI mass spectrometry imaging (MSI) requires tissue preparation strategies that preserve both tissue morphology and native molecular integrity. Mouse colon poses a particular challenge as fresh-frozen sections are mechanically fragile and frequently lose architectural definition, whereas formalin-fixed paraffin-embedded-based approaches require retrieval steps that can compromise molecular signals. Here, we present Col’RFix (Colon Rapid Fixation), a rapid, liquid-based fixation workflow optimized for transverse mouse colon tissue. Col’RFix employs short-duration cold fixation (4% paraformaldehyde at 5 °C for 10 min) combined with low-melting agarose embedding to provide mechanical support during cryosectioning. Protocol validation assessed morphology preservation, analyte diffusion, molecular coverage, and spatial localization using replicate colon sections from two high-fat diet mice analyzed across multiple MSI runs at 5, 10, and 20 μm pixel sizes, three MALDI-MSI modalities, and in positive and negative ion modes. Feasibility of biological comparison was evaluated in an independent cohort of lean (n = 3) and high-fat diet (n = 3) mice. Col’RFix enabled reproducible imaging down to 5 μm pixel size, supporting spatial lipidomics of fatty acids and multiple lipid classes with minimal delocalization. Compared to unfixed tissue sections sampled within 1 cm of the same region, Col’RFix preserved fine colon morphology, as confirmed by H&E staining, allowing precise correlation of molecular features with histological details. Spectral comparison of fixed and unfixed tissues indicated largely conserved molecular profiles, with compound-dependent signal attenuation. Together, these results establish Col’RFix as a reproducible workflow, enabling compartment-resolved molecular maps of mouse colon at 5 μm pixel size.

## Graphical abstract

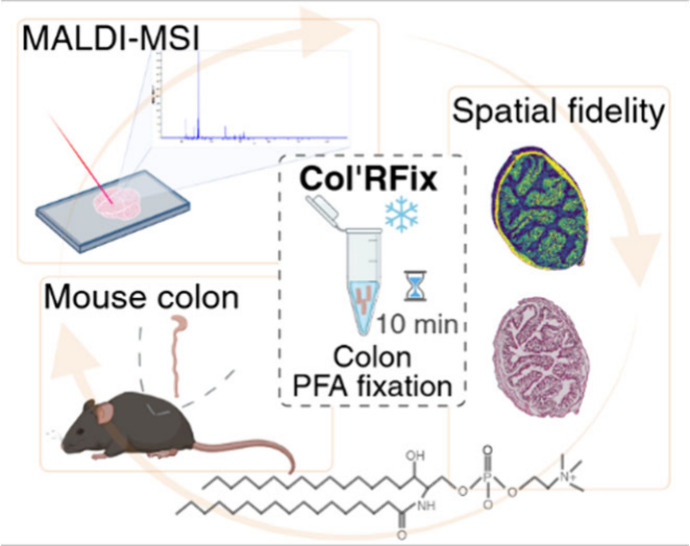

## Introduction

Mass spectrometry imaging (MSI) enables spatially resolved analysis of molecular species directly from sample surfaces, providing both molecular identity and localization. Its applications span disease pathology, biomarker and drug research,^1,2^ natural product discovery and nutrition research,^3–5^ and forensics sciences.^6^ Matrix-Assisted Laser Desorption/Ionization (MALDI)-MSI offers a versatile, label-free biomolecular imaging platform that bridges molecular specificity with spatial visualization as reviewed by Aichler & Walch (2015)^7^ and Feucherolles & Frache (2022).^8^ Recent advances in MALDI-MS imaging technology, including laser control and data acquisition methods, enable a spatial resolution approaching 1 μm.^9^ To fully match technological progress, meticulous sample preparation and proper preservation of tissue morphology are crucial to obtain reliable, high-quality imaging mass spectra.

The MSI workflow involves several critical steps, including sample collection and preservation, either as fresh-frozen (FF) or formalin-fixed paraffin-embedded (FFPE) specimens. FFPE is a standard methodology in clinical practice, providing long-term preservation for the biobanking of specimens. However, MSI of FFPE samples typically requires heating, washing, and antigen retrieval steps, which can cause analyte loss, delocalization, and modification. Despite recent advances in MSI analysis of FFPE tissue,^10,11^ fresh-frozen tissue remains preferred for preserving molecular integrity.^12^ Preserved frozen tissue samples are cryosectioned into 3–20 μm sections, mounted on conductive slides, coated with a suitable MALDI matrix, and analyzed by MSI.

The initial step of the MSI workflow is particularly challenging for certain sample types. For biological tissues, rapid collection and preservation of specimens are critical while avoiding freezing artifacts. Water-rich tissues are prone to ice crystal formation during freezing, which may cause tissue damage and complicate smooth sectioning. Fat-rich tissues, like the brain, may smear during sectioning, while fragile tissues often require additional support.

The gastrointestinal (GI) system, comprising the digestive tract, liver, pancreas, and gallbladder, plays a central role in nutrient uptake, metabolism, and waste excretion. The digestive tract hosts the largest microbial community in the mammalian body with over 10 trillion microbes, mainly bacteria, residing in the intestine,^13^ where they influence health and disease. Bidirectional interactions between the gut and organs such as brain, liver, immune, and endocrine systems have been suggested in both preclinical and clinical studies.^14–17^ This communication is largely mediated by the microbiota composition and their circulating metabolites. Due to ethical and practical constraints in human studies, preclinical murine models are commonly used to investigate intestinal molecular processes. However, the long, fragile tubular structure, narrow diameter, and rapidly postsampling degrading mucosal layer, especially due to luminal contents,^18^ pose significant challenges for MSI. The complex morphology of the intestine, including distinct layers (mucosa, muscularis externa, submucosa, and serosa) and folded epithelium forming villi and crypts, further complicates preparation. In mice, the small intestine and colon average 2.8 ± 0.3 mm and 2.9 ± 0.0 mm in diameter, respectively.^19^ Given its dense microbial population and extensive systemic interactions, the intestinal tract remains a compelling subject of study. Obviously, preserving the intestinal morphology is essential for accurate spatial molecular analysis by bioimaging technologies.

The ‘Swiss roll’ technique, introduced by Magnus (1937),^20^ described by Reilly and Kirsner in 1965 ^21^ and refined over time,^22–24^ is the preferred method for preparing rodent intestines for histological analysis. It involves longitudinal opening of the intestine, rolling, and sectioning for analysis. Applied in MSI by Guiberson et al. (2022),^25^ Zhang et al. (2022),^26^ and Ferey et al. (2024),^27^ this method enabled visualization of molecular gradients along the longitudinal axis of murine intestinal tissue. While this protocol effectively enables longitudinal mapping and maximizes tissue utilization, it does not preserve native transverse morphology. Moreover, if rolling is not performed immediately after excision, then the procedure may necessitate defrosting at the risk of molecular alterations. Alternative MSI sample preparation approaches for murine intestines include sectioning of tissue mounted in cryostat with water,^28^ carboxymethyl cellulose (CMC)^29^ or optimal cutting temperature (OCT) compound,^30^ and embedding longitudinally opened intestines in CMC (in 0.9% NaCl) for crypt-villus axis imaging.^31^ Despite these advances in handling fragile intestinal tissue, no universal protocol exists, highlighting the need to tailor the preparation methods to the research objectives.

Tissue or cell fixation is crucial in immunohistochemistry (IHC) and immunofluorescence (IF) microscopy for preserving the cellular morphology. Paraformaldehyde (PFA), commonly used at 4% in phosphate-buffered saline (PBS) at 4 °C for 4–24 h, forms covalent bonds with exposed primary amines of proteins.^32^ While some studies report that aqueous fixatives compromise the intestinal mucus layer,^33^ others show successful mucus preservation.^34^ However, none of these consider molecular preservation. Notably, microscopy studies have shown that short fixation duration (<30 min) preserved tissue structure without detrimental effects.^35^ However, cross-linking fixatives can lead to loss of signal intensity in MSI.^36^ Dannhorn et al. (2022)^12^ observed analyte loss and delocalization in 1 h PFA- and 24 h formalin-fixed liver and kidney tissues, while Kotnala et al. (2021)^37^ reported preserved lipid signatures with reduced signal intensity after 24 h of PFA fixation of human eye tissue. Similarly, Carter et al. (2016)^38^ and Bien et al. (2021)^36^ noted diminished or lost signals for phosphatidylethanolamine (PE) and phosphatidylserine (PS) in formalin-fixed lung tissue and cultured cells, respectively, with shorter fixation times (<30 min) mitigating these effects. Bien et al. (2021)^36^ also found that fixation increased cell membrane permeability, leading to molecular delocalization—effects that were mitigated by limiting fixation to less than 30 min. Despite these findings, the authors emphasized the continued need for alternative fixation strategies.^36^ Müller et al. (2023) performed PFA-fixation on cell cultures for 5 min at room temperature and saw an improved preservation of morphology at 1.5 and 5 μm pixel size.^39^ Alcohol-based fixatives (e.g., methanol, ethanol) offer an alternative to aqueous fixation by dehydrating tissues, though they may cause tissue shrinkage and hardening.^40^ Formulations like Carnoy and Methacarn preserve glycogen and nucleic acids, and buffered aqueous components can reduce fixation artifacts.^41^

To overcome the limitations of conventional tissue preparation methods for high-resolution MALDI-MSI, we developed Col’RFix (Colon Rapid Fixation) for preparing mouse colon tissue. Col’RFix addresses key challenges in sample handling by integrating rapid fixation under controlled low-temperature conditions to preserve tissue morphology and minimize diffusion artifacts and agarose embedding for structural support, enabling reproducible detection suitable for applications in spatial lipidomics. Moreover, this method fills a gap in current studies on murine intestinal tissue, which often focus on the longitudinal axis or lack the spatial resolution necessary to accurately assess morphological integrity.

## Experimental Section

### Chemicals and Reagents

Acetonitrile (ACN), methanol (MeOH) (both HPLC-grade), ethanol (EtOH), and trifluoroacetic acid (TFA) were obtained from VWR. Phosphate-buffered saline solution (PBS) was prepared in-house by dissolving 4.00 g of sodium chloride (NaCl, Supelco, 1.06404.1000), 100.75 mg of potassium chloride (KCl, Merck, P3911), 0.71 g of disodium hydrogen phosphate (Na_2_HPO_4_, Acros, 204855000), and 122.60 mg of potassium dihydrogen phosphate (KH_2_PO_4_, Sigma, 1.04873.1000) in 500 mL of distilled water. The pH was adjusted to 7.46 using 0.1 M hydrochloric acid (HCl, Merck, 100318). Paraformaldehyde, (PFA, 95%) was from Sigma (STBL1414). Distilled water was purified in-house using a Milli-Q Reference A+ system (Millipore, Søborg, Denmark). Matrix compounds included 2,5-dihydroxybenzoic acid (2,5-DHB, Aldrich, 149357–25G), 1,5-diaminonaphthalene (1,5-DAN, Sigma-Aldrich, 56451–250-MG), 2,5-dihydroxyacetophenone (2,5-DHAP, Aldrich, 760–3). Low melting point agarose was obtained from MP Biomedicals (6180–1). Sodium bicarbonate (NaHCO_3_) was from Acros (424270010), and Mayer’s Hematoxylin and Eosin Y were acquired from Sigma-Aldrich (MHS16 and HT110216, respectively). Eosin Y was acidified to 0.5% (w/v) using glacial acetic acid (Thermo Fisher, A113–50).

### Animal Study

A diet-induced obesity (DIO) mouse study was performed by Gubra ApS (Hørsholm, Denmark) using male C57BL/6JRj mice. The high-fat-diet (HFD) mice were fed the Gubra Amylin NASH (GAN) diet, comprising 40% fat, 22% fructose, and 2% cholesterol (D09100310), for 17 weeks starting at 5 weeks of age. Fat sources consisted mainly of palm oil with minor contributions from soybean oil and lard. The lean mice were fed an Altromin 1324 (Brogaarden, Lynge, Denmark) diet containing 11% fat. The animals were individually housed under a 12:12 light-dark cycle with ad libitum access to the diet. After 17 weeks, the mice were euthanized under isoflurane anesthesia, followed by cardiac puncture. The colon was excised, emptied from fecal material, cut into 2–3 cm pieces, placed on filter paper in a cassette, and frozen on crushed dry ice before storage at −70/80 °C. Freezing was required, since the animal study and MSI analysis were performed in separate facilities. Although enclosing the tissue in a cassette and using crushed dry ice provided a gentler freezing process that minimized freezing artifacts, the colonic compartment adjacent to the aqueous lumen remains particularly vulnerable to structural disruption during freezing.

### Ethics Statement

The Gubra animal facility is AAALAC accredited, and all animal experiments were conducted in accordance with local bioethical guidelines, which are compliant with internationally accepted principles for the care and use of laboratory animals. All experiments were licensed by the Danish Animal Experiment Council.

### Experimental Design

Sections from the ascending colon were used for all experiments, as detailed below. The ascending mouse colon exhibits prominent mucosal folds with well-defined crypt architecture and associated lamina propria.^42^

- **Experiment 1**. Mouse 1 (HFD): Seventeen replicates were generated, with six sections subjected to chemical fixation and eleven left unfixed. These samples were analyzed in three independent MALDI-MSI runs, each employing a different matrix, with six, six, and five sections analyzed in the first, second, and third runs, respectively, to minimize batch‑specific bias. This experiment was used to evaluate the impact of fixation on molecular and spatial integrity.
  - Pixel size: 10 μm
  - Ion modes. Negative: 1,5-DAN (MALDI), 2,5-DHAP (MALDI); Positive: 2,5-DHB (MALDI-2)
- **Experiment 2**. Mouse 2 (HFD): Nine technical replicates were prepared using Col’RFix and analyzed in nine independent MALDI-MSI runs, using three matrices to evaluate reproducibility of the fixation-based workflow.
  - Pixel size: 5 μm
  - Ion modes. Negative: 1,5-DAN (MALDI), 2,5-DHAP (MALDI); Positive: 2,5-DHB (MALDI-2)
- **Experiment 3**. Mouse 2 (HFD): Three additional consecutive technical replicates were prepared using Col’RFix and analyzed in three MALDI-MSI runs to assess compatibility of Col’RFix with different pixel sizes.
  - Pixel sizes: 5, 10, and 20 μm
  - Ion mode. Positive: 2,5-DHB (MALDI-2)
- **Experiment 4**. Mouse 3–8 (three HFD and three lean): Two technical replicates per mouse were prepared using Col’RFix and analyzed in three MALDI-MSI runs to evaluate diet-dependent molecular differences.
  - Pixel size: 5 μm
  - Ion mode. Positive: 2,5-DHB (MALDI-2)

### Colon Fixation and Embedding

Chemical fixation was performed using a solution of 4% PFA in PBS, pH 7.46. The solution was prepared by stirring at 60 °C for 2 h until clear. Volumetric and pH adjustments were made, and the solution was filtered through a 0.20 μm Phenex RC filter. The PFA solution was aliquoted into Eppendorf tubes and stored at 5 °C for a maximum of 14 days until use. Chemical fixation using a solution of Methacarn (methanol/chloroform/acetic acid (6/3/1 v/v/v)) was also explored, but several drawbacks including tissue swelling and lipid delocalization were observed, and this fixative was excluded from further analysis (data not shown). The embedding solution consisted of 2% low melting point agarose in Milli-Q water, dissolved at 80 °C and 700 rpm for 2 h. Complete dissolution was ensured to avoid residues. The solution was maintained at 40 °C and 400 rpm during the fixation and embedding procedures.

Fixation of the mouse colon was done according to the following procedure. Pieces of frozen colon tissue were transferred to the cold fixation solution (5 °C), which was immediately placed on ice in a 5 °C refrigerator for 10 min. Approximately 3.5 min before the end of fixation, the agarose solution was cooled to ∼ 25 °C under running tap water. Approximately Twenty s before the end of fixation, the colon pieces were briefly dipped in cooled melted agarose to minimize bubble formation and halt fixation. The colon sections were then placed in a cold homemade mold made from a 2 mL syringe with the top cut off. The cooled melted agarose was slowly added along the walls of the mold using a Pasteur pipet to avoid bubble formation. The colon pieces were then oriented upright at equal heights without touching each other or the mold walls using straight forceps. Once the agarose began to gel after a few minutes, further handling was avoided to prevent the holes in the gel. The mold was transferred to a stand in dry ice for rapid solidification. After approximately 2 h, the embedded tissue was pushed out of the syringe mold and stored at −80 °C. Tools were disinfected between samples. To ensure consistent anatomical origin, colon sections sampled less than 1 cm apart were either fixed following the specified procedure or left unfixed. Frozen, unfixed colon pieces were placed in a precooled mold, and a cooled agarose solution was added before completing the embedding procedure described above. Reducing fixation temperature from room temperature to 5 °C is estimated to an approximately two-fold reduction in diffusion-driven penetration over short fixation times. Considering diffusion coefficient, temperature, and contact time, fixation with the Col’RFix protocol is expected to allow the fixative to penetrate ∼ 0.11 mm into the tissue, affecting only surface colon layers.^43^ The fixation and MSI workflows are illustrated in Figure 1.

**Figure 1:**
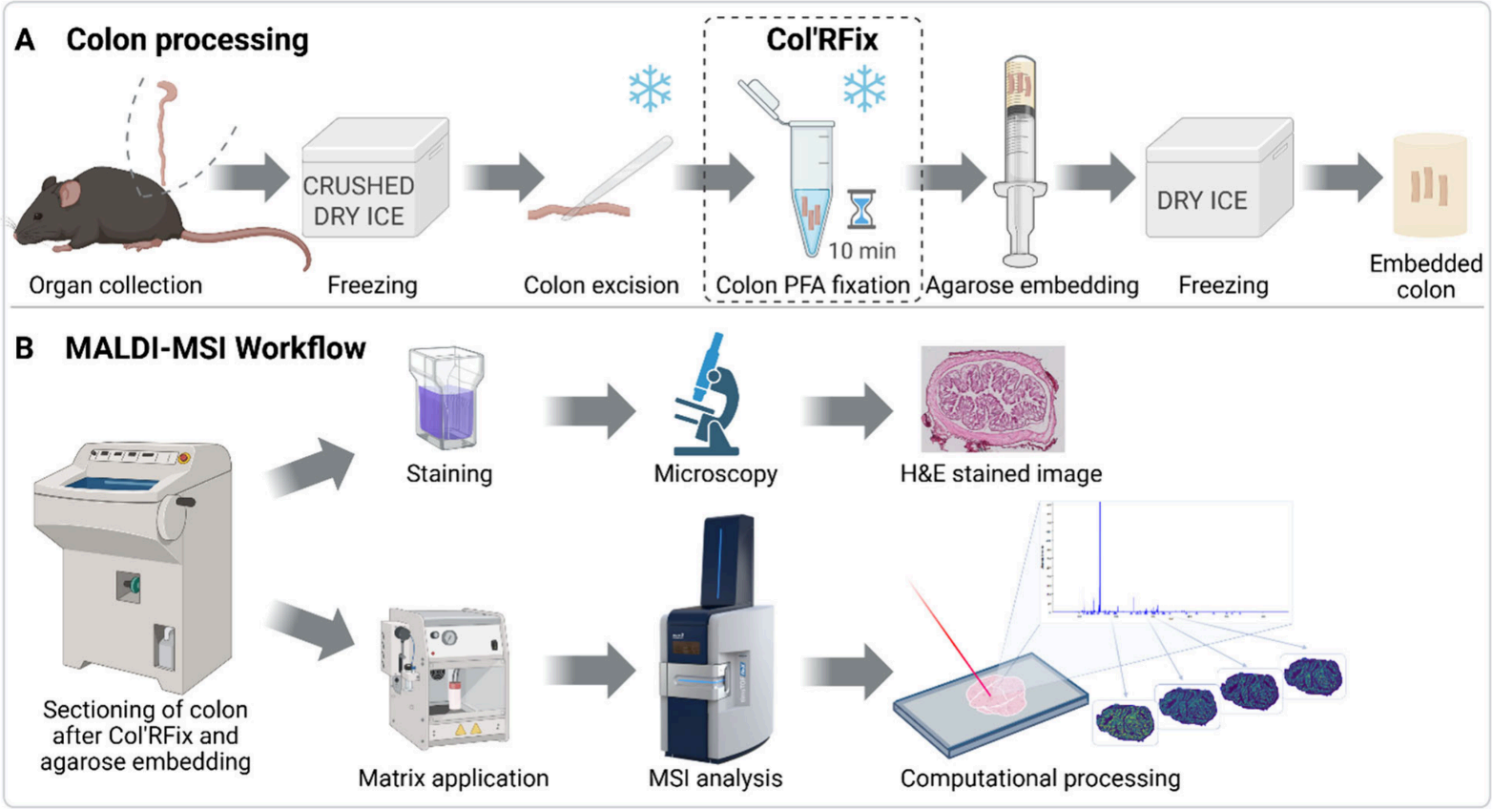
Schematic overview of the Col’RFix protocol for rapid, cold fixation of mouse colon tissue followed by agarose embedding (**A**), and MALDI-MSI workflow comprising tissue sectioning, matrix application, MSI data acquisition, and computational data analysis. These steps can be performed in parallel with histological staining and microscopy to obtain H&E images (**B**). MSI was conducted using a Bruker timsTOF fleX MALDI-2 instrument with microGRID technology (image reproduced with permission from Bruker Daltonics). Created with Biorender.com.

### MALDI-MSI and Statistics

Transverse 10 μm cross sections of fixed/unfixed and agarose-embedded mouse colons were prepared using a CM1950 cryostat (Leica Biosystems, Nussloch, Germany) at −12 °C head mount temperature and −14 °C chamber temperature. Sections were mounted on Bruker Intellislides (Bruker Daltonics, Bremen, Germany). High-fat diet mice were used throughout the study, while both lean and high-fat diet mice were included in the diet comparative analysis.

Matrix deposition was performed by using an HTX M3+ Sprayer (HTX Technologies, Carrboro, NC, USA). 2,5-DHB (10 mg/mL in 80% MeOH with 0.1% TFA) was applied at 65 °C, a flow rate of 125 μL/min, and a velocity of 1200 mm/min. 1,5-DAN (10 mg/mL in 90% ACN with 0.1% TFA) was applied at 75 °C, a flow rate of 150 μL/min, and a velocity of 1200 mm/min. 2,5-DHAP (10 mg/mL in 70% EtOH with 0.1% TFA) was applied at 75 °C, a flow rate of 100 μL/min, and a velocity of 1200 mm/min.

A timsTOF fleX instrument with MALDI-2 and microGRID (Bruker Daltonics, Bremen, Germany) was utilized and calibrated using red phosphorus. Laser pulse energy was adjusted to optimal values for both MALDI and MALDI-2 modes, with 20–200 shots applied per pixel, depending on pixel size. Data was acquired at pixel sizes of 5, 10, or 20 μm. Imaging was conducted in both negative (2,5-DHAP, MALDI and 1,5-DAN, MALDI) and positive (2,5-DHB, MALDI-2) ion modes. Data were processed using SCiLS Lab software (version 2025b Pro, Bruker Daltonics, Bremen, Germany). Data files from different MALDI-MSI runs employing the same matrix were combined into one SCiLS Lab file for each experiment. Data normalization was performed using the Root Mean Square (RMS) method, and feature finding was performed applying weak filtering, a relative intensity threshold of 0.8%, and an interval width of 12 ppm. Annotation was performed in Metaboscape (version 2025b, Bruker Daltonics, Bremen, Germany) by matching detected *m*/*z* values within a 5–10 ppm mass tolerance against the Lipid Maps and HMDB databases as well as custom target lists. RMS normalized ion density maps and mass spectra were generated in the SCiLS Lab.

Experiments 1–3 (protocol validation) were performed on technical replicates of tissue from two individual HFD mice and are reported as descriptive comparisons. Experiment 4 comprised lean (n = 3) and HFD (n = 3) mice and was analyzed by Principal Component Analysis (PCA) and Receiver Operating Characteristic (ROC) analysis for identification of discriminative molecular features. PCA and ROC analyses were performed in SCiLS Lab applying the filtered and annotated feature list obtained from feature finding comparing two dietary groups. ROC curves were generated based on all individual spectra, applying classification thresholds of 0.75 and 0.25, corresponding to a Pearson correlation significance of *p* < 0.05. PCA was performed on all individual spectra, applying weak denoising and pareto scaling and generating five principal components. Boxplots of signal intensities were generated in R using the Cardinal and ggplot2 packages based on imzML files exported from SCiLS Lab. Boxes represent Tukey hinges with the median indicated, whiskers extended to the most extreme values, and outliers defined as values beyond the whiskers.

### H&E Staining

Hematoxylin & Eosin (H&E) staining is a widely used histological technique for enhancing cellular structures and visualizing tissue morphology. A standardized staining protocol was employed on consecutive colon sections. Briefly, slide-mounted tissue sections were incubated in −20 °C methanol, followed by rinses in PBS and distilled water. Sections were stained with Mayer’s hematoxylin (5 min), immersed in 0.1% NaHCO_3_ (30 s), and counterstained with Eosin Y (2 min, 30 s) with distilled water rinses between each step. Finally, tissues were dehydrated through changes of ethanol, and coverslips were mounted. Brightfield images were acquired using an APX100 microscope (Olympus Corporation, Tokyo, Japan) at 4× optical magnification. H&E images were overlaid with ion density maps in SCiLS Lab.

## Results and Discussion

Using Col’RFix-prepared transverse mouse colon sections, we acquired MALDI-MSI data in positive and negative ion modes using three matrices to maximize molecular coverage. Across three independent MSI runs at 5 μm pixel size, we confidently annotated 126 species spanning 12 lipid classes (Table 1; Supporting Information Table S1). Going toward smaller pixel size increases anatomical specificity with detection rate depending on acquisition sensitivity. For comparison, Barone et al. (2024) reported 103 negative ion mode lipids in fresh-frozen mouse colon at 60 μm pixel size,^29^ while Ponzoni et al. (2024) annotated 83 metabolites from 275 discriminative features in rat large intestines using positive and negative ion mode MSI at 40 μm spatial resolution combined with LC-MS/MS.^30^ As expected, phosphatidic acids (PAs) were predominantly detected in negative ion mode due to efficient deprotonation, but several species were also observed in positive mode via cation adduction. Eight of the nine PAs detected in positive ion mode were also found in negative ion mode, and one PA was solely identified in positive ion mode. At the MS1 level, isobaric overlap limits unambiguous discrimination between phosphatidylcholine and phosphatidylethanolamine (PC/PE) species and limits confident assignment of the fatty acid chain length. Odd-chain fatty acids are typically low in abundance in mammalian tissues, which may aid tentative PC/PE annotation. However, as odd-chain fatty acids can originate from both microbial metabolism and dietary sources, further characterization would require higher-order structural information (e.g., MS/MS) and was beyond the scope of the present study. Representative mass spectra are provided in .

**Table 1:** Overview of Lipid Classes and Species Detected in High-Fat Diet Mouse Colon by MALDI-MSI at 5 μm Pixel Size in Positive or Negative Ion Modes Applying Different Matrices (Experiment 2, Mouse 2)^a^

Ion mode	Matrix	Laser	Molecular class^b^				
**Negative**	2,5-DHAP	MALDI	PI	PS	PG	PA	GA/GM
			24	17	8	14	7
**Negative**	1,5-DAN	MALDI	C16HyOz	C18HyOz	C20HyOz	C22HyOz	
			2	4	4	3	
**Positive**	2,5-DHB	MALDI-2	PC/PE	SM	Cer	PA	
			33	4	5	9	

a A total of 126 different species were confidently annotated within 5-10 ppm mass accuracy across all conditions. Mass spectra can be seen in .

b PI: Phosphatidylinositol, PS: Phosphatidylserine, PG: Phosphatidylglycerol, PA: Phosphatidic acid, GA/GM: Ganglioside, PC: Phosphatidylcholine, PE: Phosphatidylethanolamine, SM: Sphingomyelin, Cer: Ceramide.

### Col’RFix Preserves Colon Morphology Required for Compartment-Resolved MSI

To assess whether fixation preserves colon architecture required for compartment-resolved MSI, H&E staining was performed on transverse sections sampled <1 cm apart under fixed and unfixed conditions (mouse 1: n = 6 fixed, n = 11 unfixed). Fixed tissue (Figure 2A) retained a clear definition of the muscularis, lamina propria, basement membrane, and epithelial compartment, enabling anatomical registration of MSI signals. Localized surface epithelial discontinuities were observed, likely reflecting luminal freezing vulnerability given the aqueous luminal environment. Controlled freezing comparisons will be required to separate freezing and fixation effects on epithelial damage. In contrast, unfixed tissue (Figure 2E) exhibited a substantial loss of histological definition across layers, limiting reliable compartment assignment. Consistent results were obtained across technical replicates from experiments 1–3, supporting these observations (Supporting Information, Figures S2–S4).

**Figure 2:**
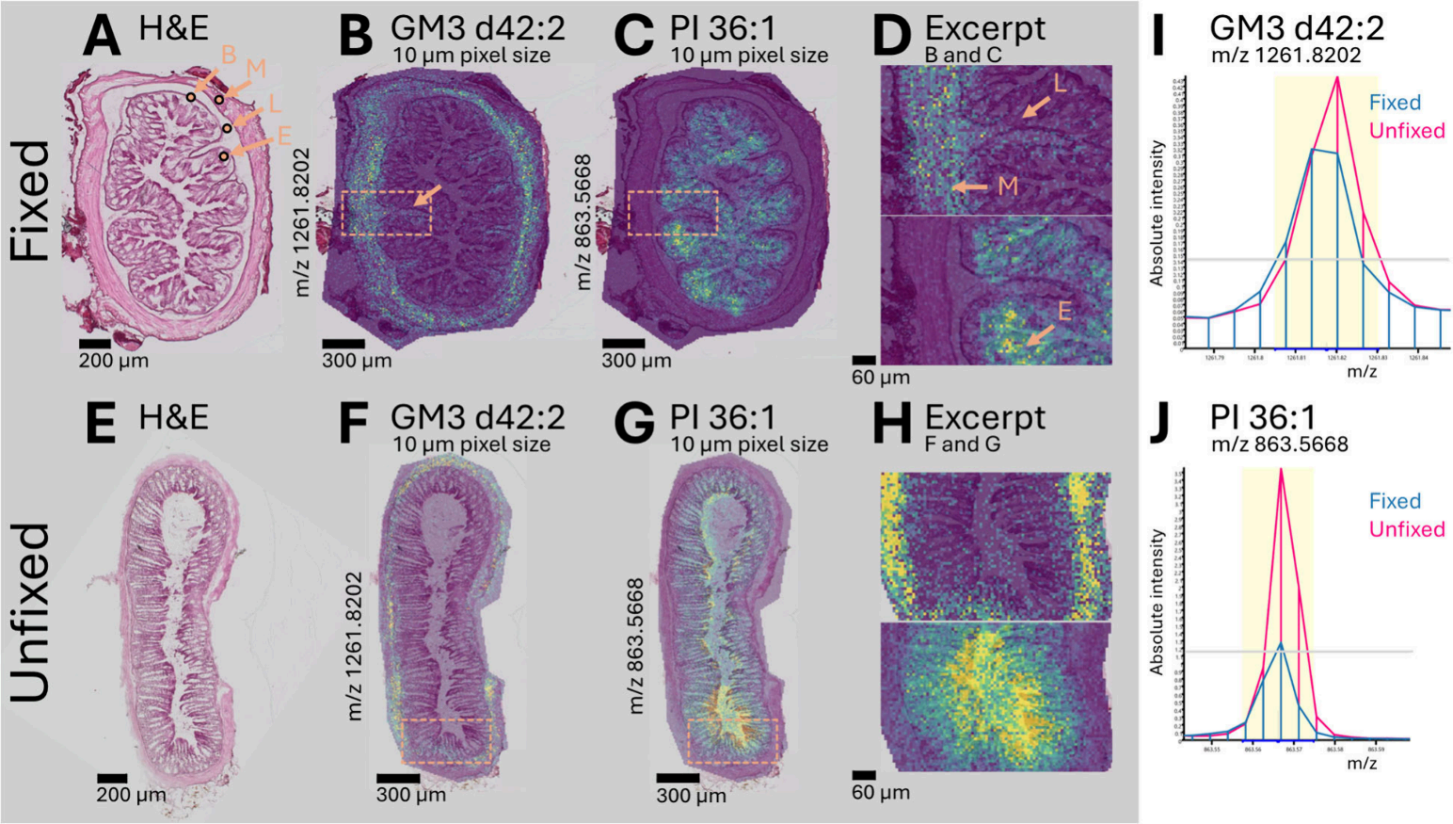
Col’RFix preserved the colon morphology required for compartment-resolved MSI (experiment 1, mouse 1). Representative brightfield H&E images show fixed (**A**) and unfixed (**E**) transverse colon sections, with basement membrane (arrow B), muscularis (arrow M), lamina propria (arrow L), and epithelia (arrow E) indicated. Ion density map overlays are shown for GM3 d42:2 (*m*/*z* 1261.8202) (**B**, **F**) and PI 36:1 (*m*/*z* 863.5668) (**C**, **G**), acquired at a 10 μm pixel size using 2,5-DHAP (MALDI, negative ion mode). Enlarged regions (**D**, **H**) highlight improved anatomical definition and compartment localization in fixed tissue with GM3 d42:2 localized in muscle (arrow M) and lamina propria (arrow L) and PI 36:1 localized in epithelia (arrow E). Representative spectra from fixed (blue) and unfixed (pink) regions (**I**, **J**) show overall conservation of major features, consistent with preserved molecular profiles and compound-dependent signal attenuation following fixation. Scale bars: Ion density maps 300/60 μm; H&E 200 μm.

To link morphology to molecular localization, H&E images were overlaid with RMS-normalized ion density maps acquired at 10 μm pixel size (2,5-DHAP, negative mode). GM3 d42:2 (*m*/*z* 1261.8202) localized primarily to the muscularis and lamina propria in fixed tissue (Figure 2B), whereas the unfixed section showed reduced structural correspondence and apparent confinement of signal to the muscle layer (Figure 2F). PI 36:1 (*m*/*z* 863.5668) outlined crypt-like epithelial features in fixed tissue (Figure 2C), which were less distinct in unfixed tissue (Figure 2G). Mass spectra from matched regions (Figure 2I-J) showed a compound-specific reduction in signal intensity following fixation but broadly conserved molecular profiles between conditions, supporting the idea that Col’RFix maintains molecular integrity while enabling morphologically interpretable imaging.

### Fixation Improves Spatial Fidelity of Lipid Localization

Cold, short-duration PFA fixation was chosen to stabilize architecture while limiting diffusion; therefore, next we assessed whether preserved morphology translated into improved spatial fidelity of lipid distributions. For the selected lipid classes, spectral comparison of features revealed the presence of 150 species in both unfixed and fixed tissue, although some species were below the intensity threshold (Supporting Information Table S2). In positive ion mode (2,5-DHB, MALDI-2, 10 μm pixel size), structured, compartment-consistent localization in fixed tissue, including epithelial crypt-like organization, was observed, whereas unfixed tissue exhibited diffuse and less anatomically interpretable distributions (Figure 3A-F). In fixed tissue, SM d38:2 (*m*/*z* 793.5601) localized predominantly to the muscularis, basement membrane, and epithelial regions and was absent from the lamina propria. In contrast, unfixed tissue exhibited a diffuse and less structured distribution. A similar trend was observed for PE P-36:2/PC P-33:2 (*m*/*z* = 728.5595), which was localized in the epithelial layer of the colon. Overlay images of SM d38:2 and PE P-36:2/PC P-33:2 in fixed (Figure 3C) and unfixed (Figure 3F) outlined sections further demonstrated that fixation preserved fine morphological detail, enabling clear delineation of colon layers, while unfixed tissue lacked defined structural detail. For these representative ions, signal intensity was maintained or only modestly reduced after fixation, although attenuation was compound-dependent across the broader data set.

**Figure 3:**
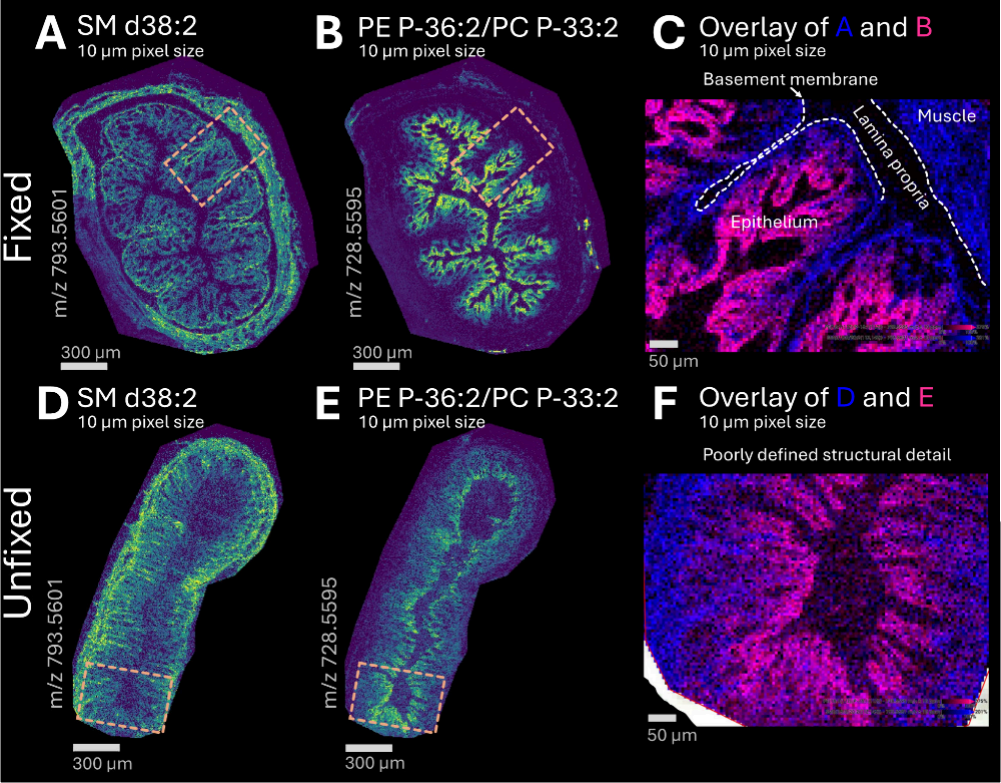
Col’RFix preserved complex colon tissue architecture for high-spatial resolution MSI, as shown by ion density maps of transverse colon cross sections (experiment 1, mouse 1). Fixed (**A**, **B**) and unfixed (**D**, **E**) tissues were analyzed in positive ion mode at 10 μm pixel size using 2,5-DHB (MALDI-2). **A** and **D** show the distribution of SM d38:2 (*m*/*z* 793.5601), while **B** and **E** depict PE P-36:2/PC P-33:2 (*m*/*z* 728.5595). Scale bar: 300 μm. Overlays of SM d38:2 (blue) and PE P-36:2/PC P-33:2 (pink) in the outlined area of fixed (**C**) and unfixed (**F**) tissue highlight the improved structural definition achieved with Col’RFix, enabling delineation of colon layers. Scale bar: 50 μm.

### Minimal Fixation-Specific Delocalization

To evaluate potential analyte delocalization, ion density maps of representative species across fatty acids and major lipid classes acquired at 5 μm pixel size and different MALDI-MSI modalities were examined. Across compound classes listed in Table 1, we did not observe fixation-specific diffusion patterns, such as systematic enrichment at tissue edges or boundary blurring beyond the native compartment distributions (Figure 4). As indicated in the magnified excerpts, PI 36:4 (*m*/*z* 857.5194) and PG 34:1 (*m*/*z* 747.5167) were predominantly localized to the epithelial layers, whereas PS 44:8 (*m*/*z* 886.5000) and PA 40:4 (*m*/*z* 751.5266) were distributed across both muscular and epithelial layers. GM3 d40:1 (*m*/*z* 1235.8022) was highly abundant in muscular tissue and the lamina propria. Fatty acids C_22_H_32_O_2_, C_20_H_32_O_2_, C_18_H_34_O_2_, and C_16_H_32_O_2_ (*m*/*z* 327.2333, 303.2336, 281.2484, and 255.2334) were broadly distributed in both muscle and epithelial compartments. PE 38:5/PC 35:5, PE 40:3/PC 37:3, and PE 44:4/PC 41:4 species (*m*/*z* = 766.5400, 798.6013, and 852.6480), SM d35:0 (*m*/*z* = 757.5593), and PA 38:5 (*m*/*z* = 761.4518) exhibited variable localization across epithelial and muscular regions. The protocol did not resolve triglycerides in either unfixed or fixed colon tissue. Agarose embedding has previously been shown not to induce molecular delocalization or alter the number of annotated lipid species.^44^ However, the use of a fully aqueous embedding medium may be incompatible with the retention of neutral, apolar triglycerides, which could contribute to their delocalization in colon tissue (Supporting Information, Figure S5).

**Figure 4:**
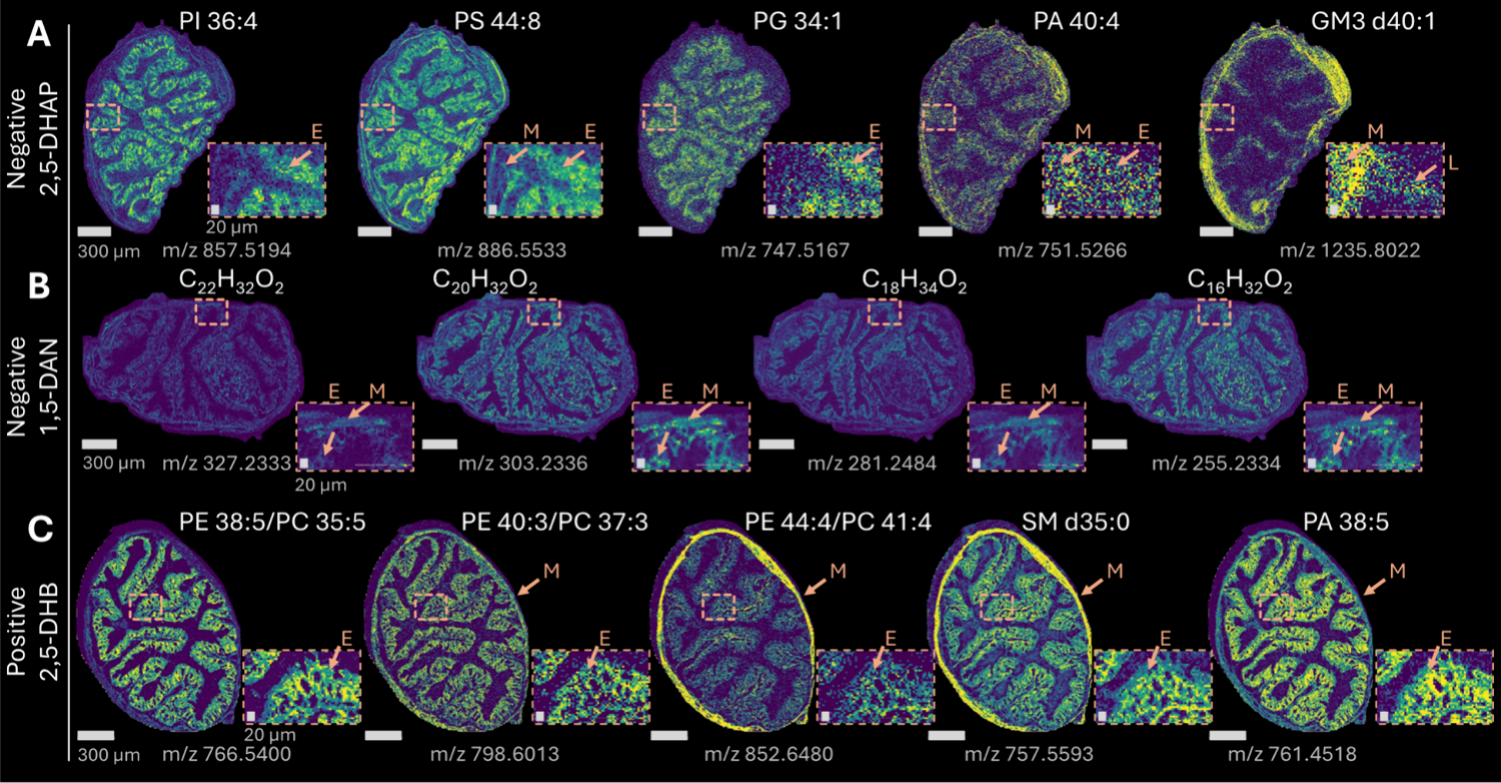
Col’RFix method preserved tissue morphology and biomolecular distribution with minimal diffusion, as demonstrated by ion density maps of fixed transverse colon cross sections acquired at 5 μm pixel size representing different compound classes (mouse 2). Row **A** (left to right) displays PI 36:4, PS 44:8, PG 34:1, PA 40:4, and GM3 d40:1 acquired in negative ion mode (2,5-DHAP, MALDI). Row **B** (left to right) shows fatty acids C_22_H_32_O_2_, C_20_H_32_O_2_, C_18_H_34_O_2_, and C_16_H_32_O_2_ acquired in negative ion mode (1,5-DAN, MALDI). Row **C** (left to right) presents PE/PC (3 species), SM d35:0, and PA 38:5 acquired in positive ion mode (2,5-DHB, MALDI-2). Outlined areas provide a magnified view of signal distribution in colon compartments; muscle (M), epithelia (E), lamina propria (L). Scale bars: 300 μm/20 μm.

For some species, uneven intensity patterns were observed in both fixed and unfixed tissues (Figure 5). While the underlying cause cannot be unambiguously determined, these patterns are consistent with solvent-driven redistribution during wet matrix application, potentially arising from variations in tissue thickness or incomplete sealing at tissue edges or with altered local ionization environment, potentially associated with triglyceride accumulation. This is illustrated in ion density maps of PI species acquired at a pixel size of 10 μm using a 2,5-DHAP matrix in negative ion mode (Figure 5). Mean regional fold-change analysis showed that fixation was associated with a marked lower-to-upper intensity contrast in both muscle and epithelial compartments, with fixed tissue showing an approximately 2.3-fold higher intensity in the lower region compared to an ∼ 1.6-fold higher intensity in unfixed tissue (Figure 5E). This observation aligns with previous findings by Bien et al. (2021),^36^ who reported similar diffusion patterns in fixed and unfixed cell cultures, and is further in line with expectations based on the limited penetration depth of the fixative, which is sufficient to stabilize surface tissue layers.

**Figure 5:**
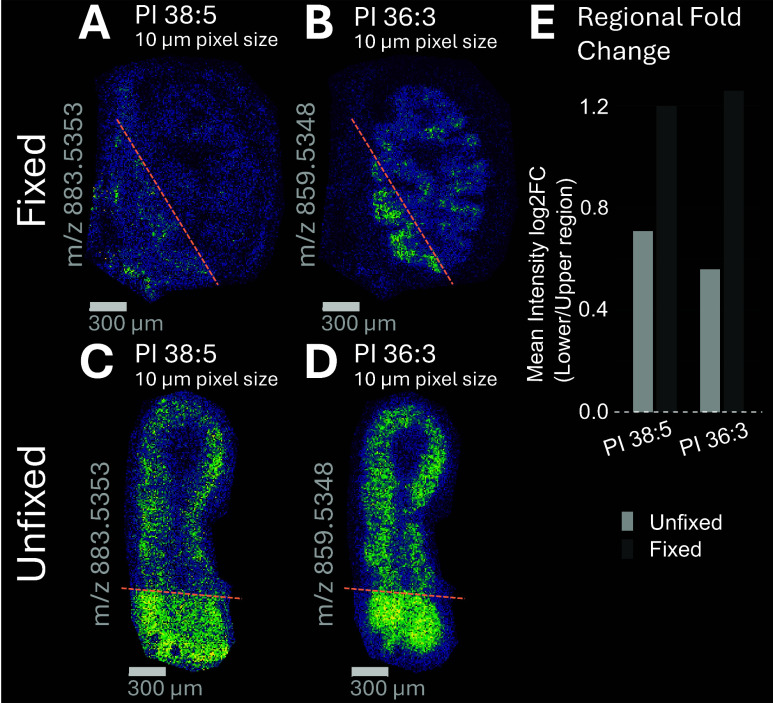
Minor intensity heterogeneity was observed in both fixed and unfixed transverse colon cross sections, attributable to wet matrix deposition or altered local ionization environment (experiment 1, mouse 1). Ion density maps, acquired at a 10 μm pixel size using 2,5-DHAP (MALDI, negative ion mode), illustrate this effect, with regions below the dashed lines indicating areas of increased signal intensity. **A** and **C** show the spatial distribution of PI 38:5 (*m*/*z* 883.5353) in fixed and unfixed tissue, respectively, while **B** and **D** display PI 36:3 (*m*/*z* 859.5348) under the same conditions. Scale bar: 300 μm. Barplot of mean regional intensity fold-change of region below dashed line (lower) vs above dashed line (upper) showing ∼ 2.3-fold higher intensity in the lower region in fixed tissue compared to ∼ 1.6-fold higher intensity in unfixed tissue (**E**).

### Col’RFix Supports 5 μm MALDI-MSI with Expected Intensity Trade-Offs

The level of spatial detail achieved with microGRID was identified by analyzing consecutive sections of fixed colon coated with a DHB matrix in positive ion mode with MALDI-2 at pixel sizes of 5 and 20 μm, respectively. Using microGRID-enabled acquisition, lipid distributions could be resolved at 5 μm pixel size with markedly improved visualization of fine anatomical features compared to 20 μm (Figure 6A-B). MSI signals were assigned to SM d36:1 (*m*/*z* 731.6058, magenta), PA 38:5 (*m*/*z* 745.4770, orange), and PC 36:2/PE 39:2 (*m*/*z* 786.5976, green). The distribution of these compounds varied across different morphological regions of the colon. Specifically, SM d36:1 was predominantly found in muscular layers and basement membrane, PA 38:5 was present in muscular tissue and showed stronger intensity in the epithelial layer, while PC 36:2/PE 39:2 was located in tissue adjacent to the lumen. At 20 μm pixel size, the image lacked sufficient detail to resolve lumen structures and fine connective features such as the basement membrane. Overlay with H&E confirmed correspondence between lipid localization and histological compartments as illustrated by SM d36:1 (*m*/*z* 769.5605) acquired at 5 μm pixel size (Figure 6C). SM d36:1 was strongly represented in the muscularis and also detectable within the epithelial compartment. As expected, reducing the pixel size decreased the absolute signal intensity due to a smaller effective sampling area per pixel (Figure 6D). This is consistent with previous observations in high-resolution MALDI-MSI.^45^ RMS normalization compensates for these effects, enabling a comparison across resolutions.

**Figure 6:**
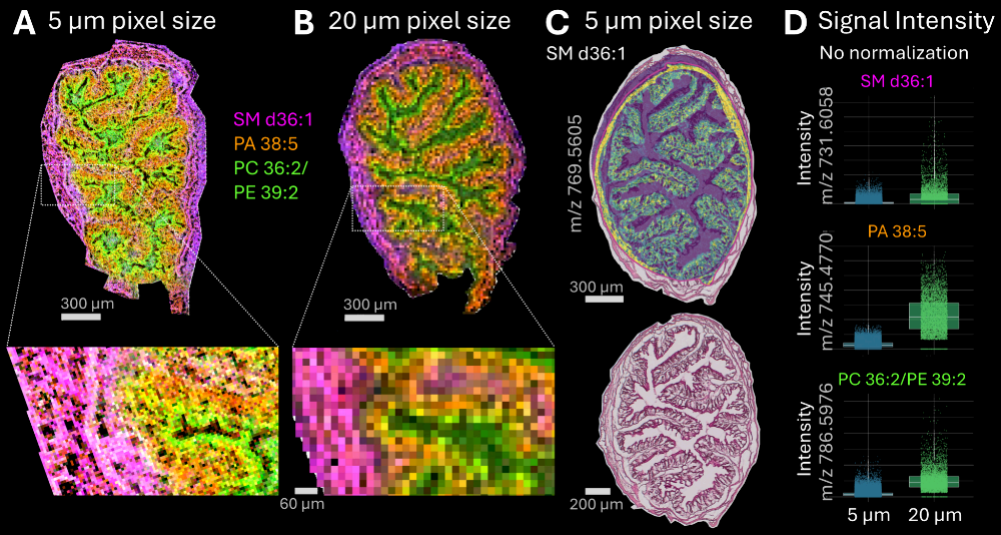
Improved Col’RFix sample preparation for high-spatial resolution MSI enabled visualization of mouse colon fine details as seen by ion density maps of transverse colon cross sections acquired using 2,5-DHB (MALDI-2, positive ion mode) at **A**) 5 μm pixel size and **B**) 20 μm pixel size with enlarged excerpts showing detailed localization (experiment 3, mouse 2). The signals were assigned to SM d36:1 (*m*/*z* = 731.6058, magenta), PA 38:5 (*m*/*z* = 745.4770, orange), and PC 36:2/PE 39:2 (*m*/*z* = 786.5976, green). Scale bars: 300/60 μm. **C**) An overlay of the ion density map of SM d36:1 (*m*/*z* 769.5605), acquired at 5 μm pixel size with the corresponding H&E image showed colocalization to colon morphological structure. Scale bar H&E: 200 μm. **D**) Boxplots of non-normalized signal intensities of SM d36:1 (top), PA 38:5 (middle), and PC 36:2/PE 39:2 (bottom) at 5 μm pixel size (blue bars) and 20 μm pixel size (green bars).

### Feasibility of Diet Group Comparison

Finally, we tested whether Col’RFix-prepared colon tissue supported comparative MSI analysis between lean and high-fat diet mice for data acquired at 5 μm pixel size. PCA showed differentiation of dietary groups, with molecular species driving the variation located outside a 50% error ellipse. ROC analysis identified features with modest discriminatory power, exemplified by SM d42:3 (*m*/*z* 833.6505) with an AUC of 0.66 (Figure 7). Features with no discrimination between dietary groups included PA 38:5 (*m*/*z* 723.4955) with an AUC of 0.50. While an AUC value of 0.66 does not reflect strong classification performance, these examples demonstrate the feasibility of group comparison.

**Figure 7:**
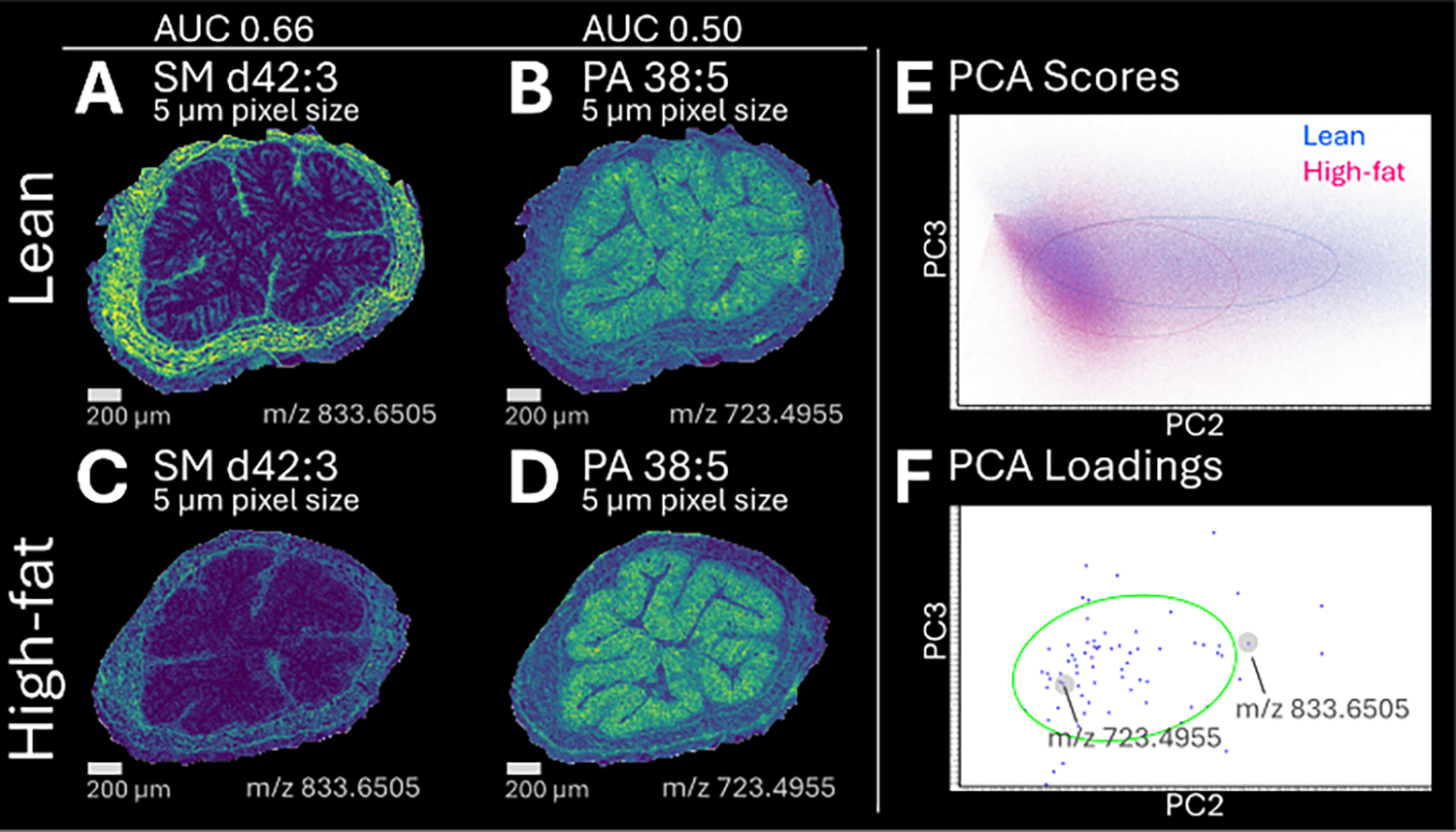
Molecular features distinguishing lean (*n* = 3) and high-fat (*n* = 3) diet groups were identified based on PCA and ROC comparative analysis of colon tissue following fixation with the Col’RFix protocol (experiment 4, mice 3–8). Ion density maps illustrate representative features in lean (**A**, **B**) or high-fat (**C**, **D**) diet tissue acquired at 5 μm pixel size using 2,5-DHB (MALDI-2, positive ion mode). Examples illustrating varying degrees of molecular discrimination between dietary conditions are SM d42:3 (*m*/*z* 833.6505, AUC of 0.66), which was enriched in lean tissue (**A**, **C**), and PA 38:5 (*m*/*z* 723.4955, AUC of 0.5), which had no discriminatory power between diets (**B**, **D**). Scale bar: 200 μm. PCA scores and loading plots show differentiation of lean and high-fat diet mice with molecular species driving the variation, showing outside a 50% error ellipse (**E**, **F**).

As illustrated in Figure 6, lower pixel size resulted in decreased absolute signal intensity, and as shown in Figure 2, fixation contributed to compound-dependent signal attenuation, increasing the risk of losing low-abundance molecular species near the detection threshold. Careful optimization of acquisition parameters and sample preparation is essential to balance the spatial resolution with molecular sensitivity. Within these constraints, Col’RFix enabled high-resolution MSI with precise localization of molecular species across distinct colon layers, providing spatial specificity relevant for investigating region-specific metabolism, molecular uptake, and biotransformation processes within the gastrointestinal tract.

## Conclusions

High-spatial-resolution MALDI-MSI places stringent requirements on sample preparation, particularly for mechanically fragile and morphologically complex tissues such as the mouse colon. Previous studies have emphasized that conventional fixation procedures can compromise molecular integrity, introduce molecular delocalization, or result in signal loss depending on fixation conditions and duration.^12,36^

In this study, we developed Col’RFix, a rapid, cold fixation workflow based on 4% paraformaldehyde fixation at 5 °C for 10 min combined with low-melting agarose embedding, to support transverse cryosectioning of mouse colon tissue. Protocol validation using multiple tissue sections from two individual high-fat diet mice, analyzed across 15 MALDI-MSI runs, three matrices, and pixel sizes of 5, 10, and 20 μm, demonstrated that Col’RFix supports MSI at 5 μm pixel size while maintaining structural interpretability. Comparable numbers of annotated lipid species were obtained in fixed and unfixed colon tissue across the lipid classes examined. At 5 μm pixel size, 126 lipid species spanning 12 lipid classes were annotated in the fixed colon tissue.

Histological assessment by H&E staining showed that Col’RFix preserved colonic layer architecture sufficiently to enable anatomical registration of MSI data, whereas matched unfixed sections exhibited reduced histological definition, limiting reliable compartment assignment. Given the expected penetration depth of the cold fixative, stabilization was primarily confined to surface tissue layers. As lower temperatures generally slow diffusion-driven molecular processes,^46^ fixation at reduced temperature is anticipated to limit molecular diffusion, although not directly evaluated in the present study. Prior studies employing short-duration fixation at room temperature in organoids and cell cultures have reported improved morphological preservation, while the potential effect on molecular diffusion was not discussed.^39,47^ Overlays of ion density maps with histological images further demonstrated improved spatial fidelity of lipid localization, enabling alignment of molecular distributions with defined anatomical features, such as epithelium, basement membrane, lamina propria, and muscular layers.

Assessment of analyte delocalization across lipid classes and acquisition modalities indicated no fixation-specific diffusion patterns for the lipid classes examined, although molecular delocalization is a known limitation of current prolonged liquid-based fixation methods.^12,37,38,36^ Triglycerides could not be spatially resolved in either a fixed or unfixed colon, suggesting that neutral, apolar lipid species are not retained under aqueous embedding conditions. Spectral comparison of fixed and unfixed tissue showed compound-dependent signal attenuation following fixation. Prior studies have shown that short fixation durations can preserve molecular profiles while stabilizing tissue morphology,^40^ consistent with data presented in this study. Spatial intensity heterogeneity observed in both fixed and unfixed tissue was associated with wet matrix application or to a locally altered ionization environment, rather than to fixation-induced delocalization.

At 5 μm pixel size, microGRID-enabled acquisition provided improved visualization of fine anatomical features compared to 20 μm, accompanied by an expected reduction in absolute signal intensity. This trade-off is consistent with previous high-resolution MALDI-MSI studies, reporting decreased signal intensity at small pixel sizes,^45^ highlighting the need to balance spatial resolution with molecular sensitivity.

In an independent diet feasibility experiment using lean (n = 3) and high-fat (n = 3) diet mice, MSI data acquired at 5 μm pixel size using 2,5-DHB (MALDI-2) supported exploratory comparative analysis. PCA and ROC analysis identified molecular features with modest discriminatory performance between dietary groups, demonstrating compatibility of Col’RFix-prepared tissue with comparative MSI workflows.

Previous studies have demonstrated that intestinal tissue from both mice and rats can be processed without fixation prior to MSI.^29,30,28,31^ However, anatomical differences between species have practical implications for sample handling. The rat colon, with a diameter exceeding 8 mm, provides a greater mechanical stability during sectioning than the significantly narrower mouse colon, which may contribute to improved preservation of tissue architecture reported in several studies.^28,30,31^ Barone et al. (2024)^29^ concluded that MSI of unfixed mouse colon tissue at 60 μm spatial resolution did not enable colocalization of lipids with specific anatomical compartments,^29^ a limitation that may reflect both low spatial resolution and compromised structural integrity.

While the ‘Swiss roll’ technique remains valuable for assessing longitudinal molecular gradients in intestinal tissue, Col’RFix offers advantages for spatially resolved molecular imaging of transverse colon cross sections. In comparison to published intestinal MSI studies performed at coarser spatial resolution, Col’RFix supports MSI down to 5 μm pixel size while retaining transverse architectural interpretability required for compartment-resolved molecular mapping.

Although matrix sublimation was not applied in this study, it represents a dry matrix deposition strategy that minimizes solvent-driven analyte migration and is expected to be fully compatible with the Col’RFix workflow. In addition, the protocol is anticipated to be adaptable to other MS ionization modalities, including DESI^48^ and t-MALDI,^9^ offering broader applicability in spatial lipidomics.

## Supplementary Material







## Notes

MALDI-MSI mass spectra acquired using three acquisition modalities, ion density maps from technical replicates, ion density maps illustrating triglyceride delocalization, tables listing detected *m*/*z* features, lipid annotation, and assignment details (PDF)
Table listing detected *m*/*z* features, lipid annotation, and assignment details (XLSX)
Table listing detected *m*/*z* features, lipid annotation, and assignment details (XLSX)
