# Supplementary material for: Rapid Cold Fixation of Mouse Colon (Col’RFix) Enables High-Resolution Mass Spectrometry Imaging
Source: Anal Chem. 2026 Apr 13;98(16):11652–61. doi: 10.1021/acs.analchem.5c04968 (PMC13130168; doi:10.1021/acs.analchem.5c04968)
Supplement: Supplementary file 1 [file ac5c04968_si_001.pdf]

Supporting Information

Rapid Cold Fixation of Mouse Colon (Col'RFix) Enables High-Resolution Mass Spectrometry Imaging

Sabina H. Skov[1,2]*, Henrik M. Jensen[2], Ole N. Jensen[1]*

[1]Department of Biochemistry and Molecular Biology,

University of Southern Denmark, DK-5230 Odense M, Denmark.

[2]Advanced Analytical R&D, Health & Biosciences,

International N&H Denmark ApS (IFF), DK-8220 Brabrand, Denmark.

***Corresponding authors:**

Ole N. Jensen, Jenseno@bmb.sdu.dk

Sabina H. Skov, sabina.holm.skov@iff.com

ORCID:

Sabina Holm Skov: 0009-0001-6957-7014

Henrik Max Jensen: 0009-0002-9898-6850

Ole N. Jensen: 0000-0003-1862-8528

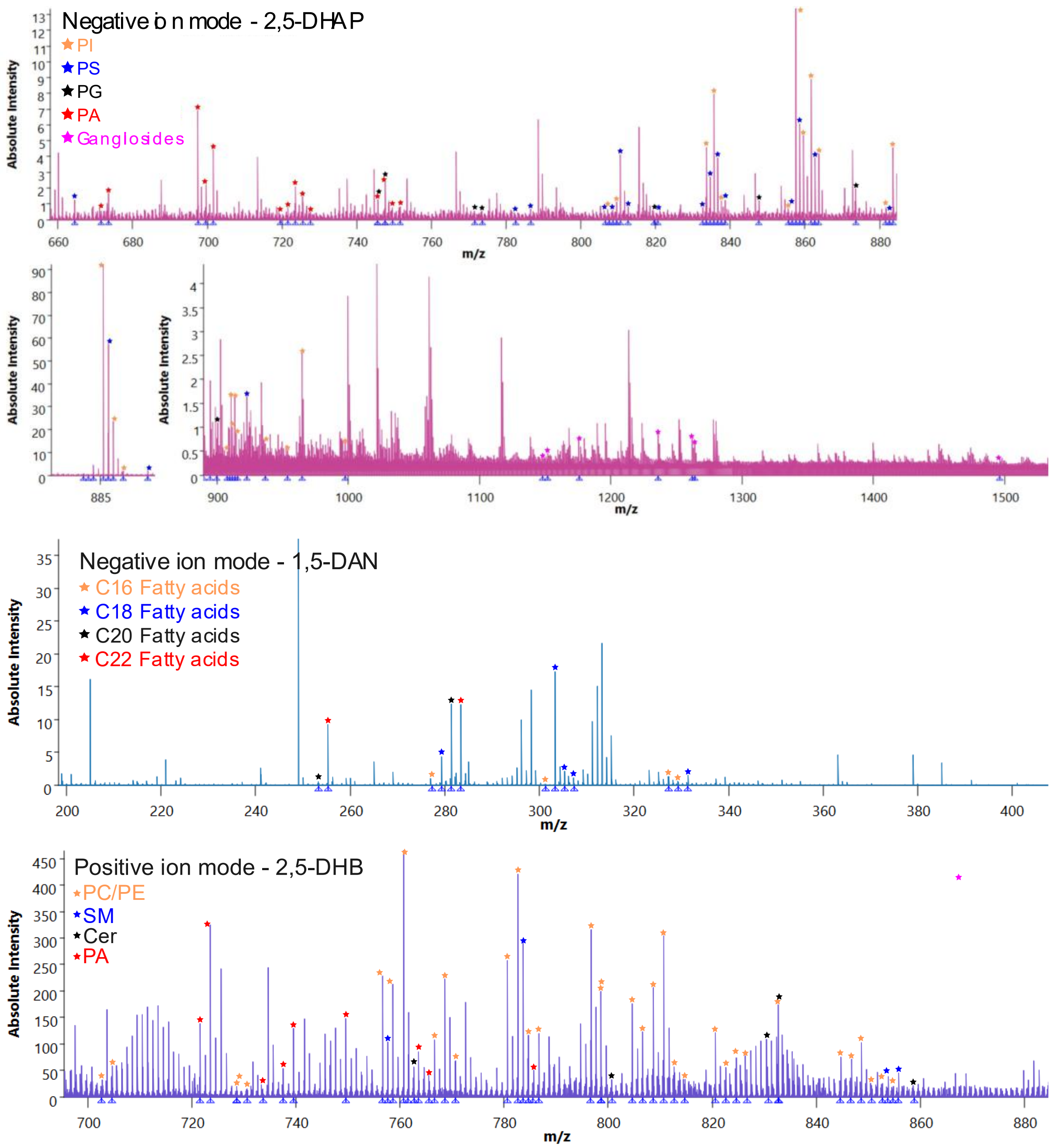

SI Figure S1. Mass spectra acquired using three MSI methods revealed lipid species annotated within a mass accuracy of 5-10 ppm across distinct m/z ranges. Spectra are colour-coded by ionization mode and matrix: Pink for negative mode with 2,5-DHAP (MALDI), blue for negative mode with 1,5-DAN (MALDI), and purple for positive mode with 2,5-DHB (MALDI-2). Annotated species are marked with coloured stars, each representing a distinct molecular class, highlighting the complementary detection profiles enabled by the different MSI conditions.

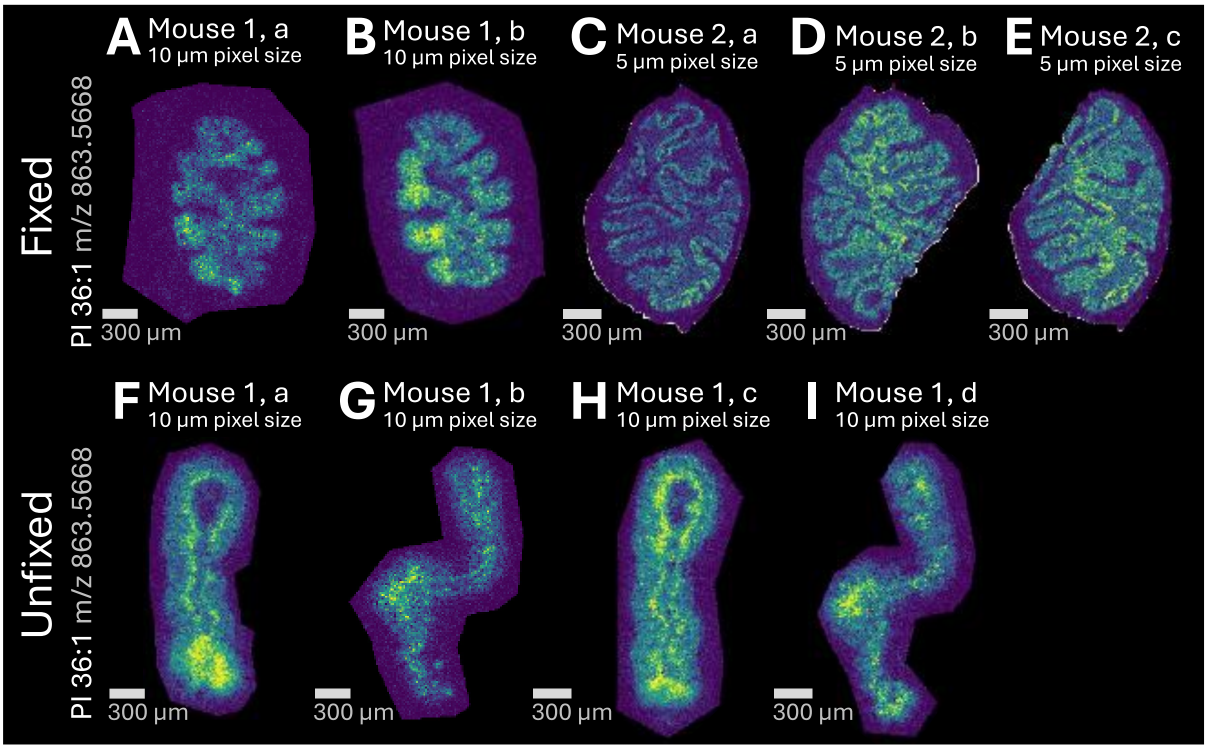

SI Figure S2. Ion density maps of PI 36:1 (m/z 863.5668) acquired from replicate mouse colon tissue sections that were either fixed or left unfixed. Sections from mouse 1 and mouse 2 were analyzed by MALDI-MSI in negative ion mode using 2,5-DHAP as matrix, at pixel sizes of 5 µm and 10 µm (MALDI). Scale bar: 300 µm.

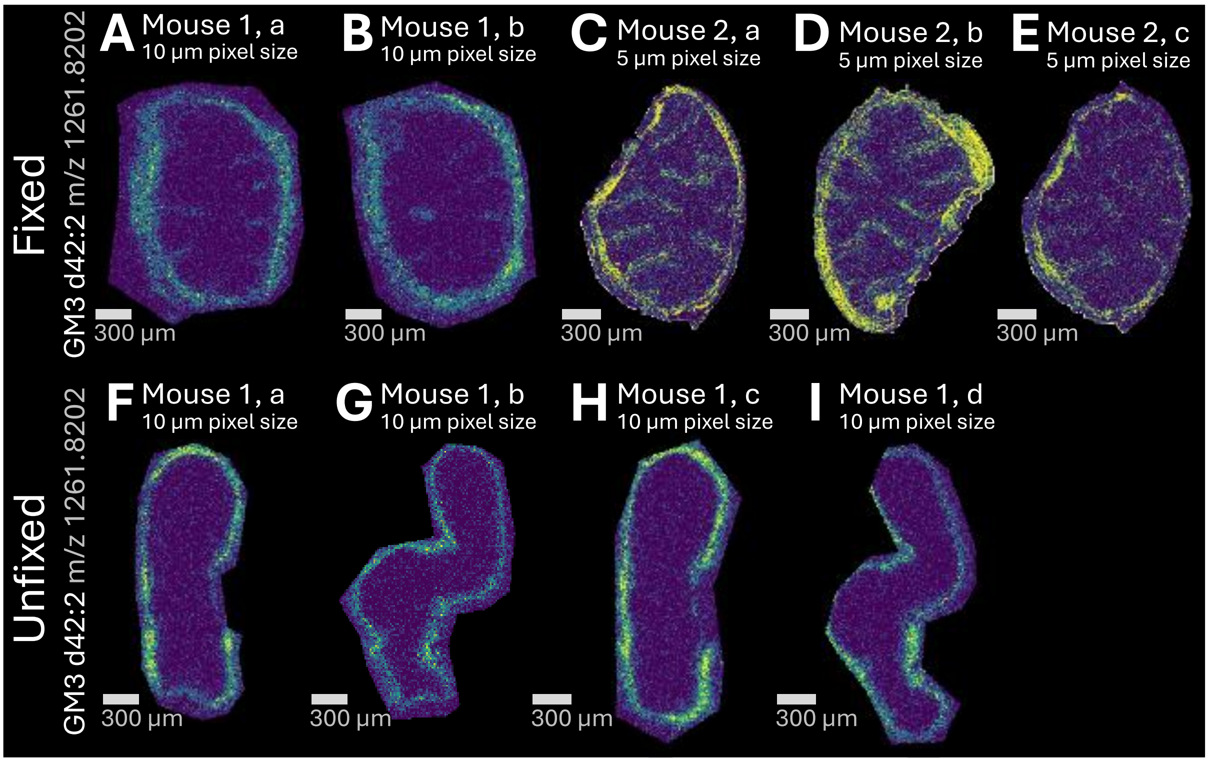

SI Figure S3. Ion density maps of GM3 d42:2 (m/z 1261.8202) acquired from replicate mouse colon tissue sections that were either fixed or left unfixed. Sections from mouse 1 and mouse 2 were analyzed by MALDI-MSI in negative ion mode using 2,5-DHAP as matrix, at pixel sizes of 5 µm and 10 µm (MALDI). Scale bar: 300 µm.

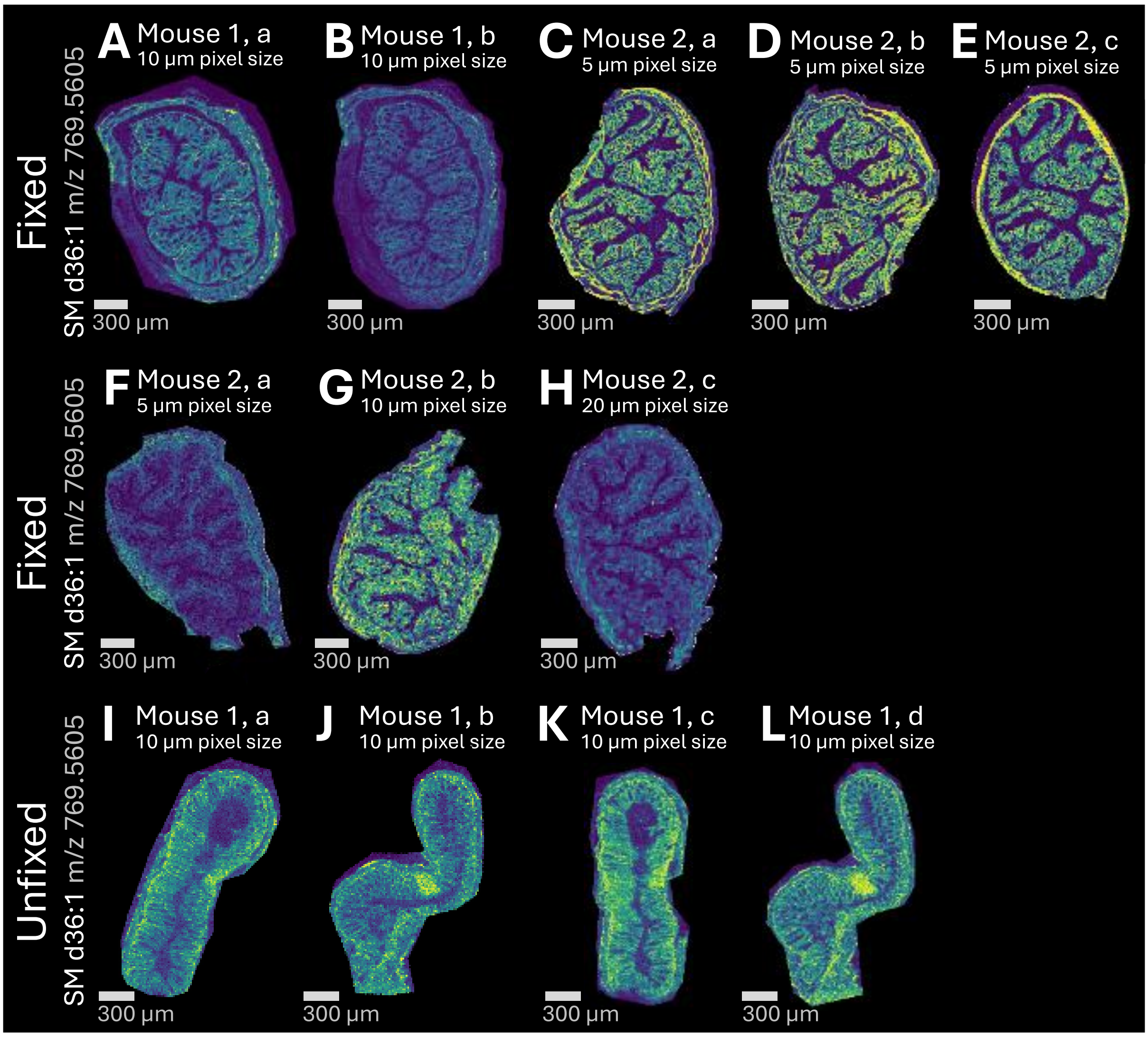

SI Figure S4. Ion density maps of SM d36:1 (m/z 769.5605) acquired from replicate mouse colon tissue sections that were either fixed or left unfixed. Sections from mouse 1 and mouse 2 were analyzed by MALDI-MSI in positive ion mode using 2,5-DHB as matrix, at pixel sizes of 5 µm, 10 µm, and 20 µm (MALDI-2). Scale bar: 300 µm.

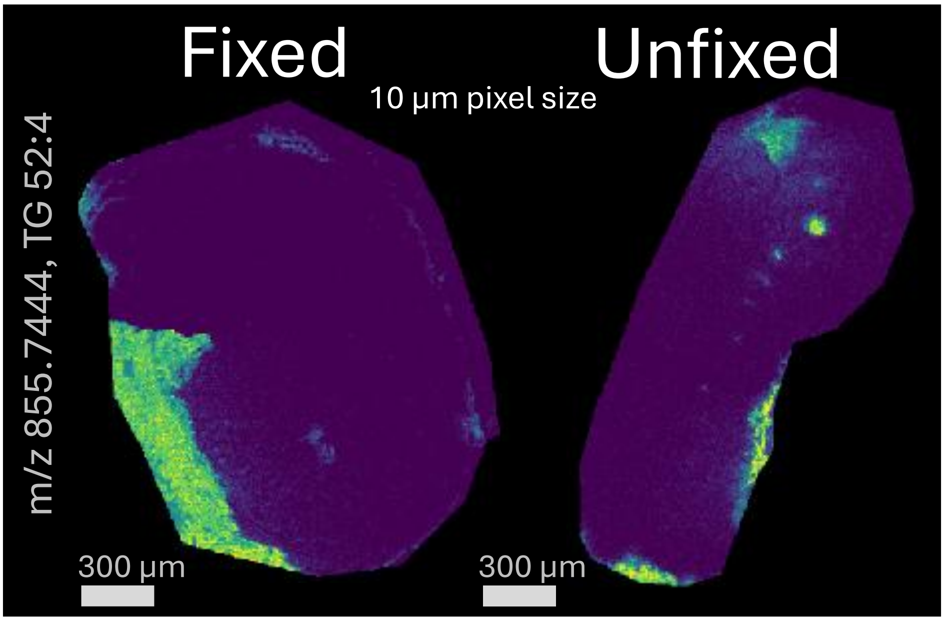

SI Figure S5. Delocalization of triglycerides exemplified by TG 52:4 (m/z 855.7444) was observed for both fixed and unfixed colon tissue, reflecting lack of retention during agarose embedding (Mouse 1, 2,5-DHB, MALDI-2).
